# Realized thermal niche approach eliminates temperature bias in bioenergetic model estimates

**DOI:** 10.1002/ece3.10974

**Published:** 2024-02-14

**Authors:** Silviya V. Ivanova, Aaron T. Fisk, Timothy B. Johnson

**Affiliations:** ^1^ Great Lakes Institute for Environmental Research University of Windsor Windsor Ontario Canada; ^2^ School of the Environment University of Windsor Windsor Ontario Canada; ^3^ Ontario Ministry of Natural Resources and Forestry Picton Ontario Canada

**Keywords:** behavior, bioenergetics, model bias, prey consumption, realized niche, thermal occupancy

## Abstract

Bioenergetics models estimate ectotherm growth, production, and prey consumption – all key for effective ecosystem management during changing global temperatures. Based on species‐specific allometric and thermodynamic relationships, these models typically use the species' lab‐derived optimum temperatures (physiological optimum) as opposed to empirical field data (realized thermal niche) that reflect actual thermal experience. Yet, dynamic behavioral thermoregulation mediated by biotic and abiotic interactions may provide substantial divergence between physiological optimum and realized thermal niche temperatures to significantly bias model outcomes. Here, using the Wisconsin bioenergetics model and in‐situ year‐round temperature data, we tested the two approaches and compared the maximum attainable lifetime weight and lifetime prey consumption estimates for two salmonid species with differing life histories. We demonstrate that using the realized thermal niche is the better approach because it eliminates significant biases in estimates produced by the physiological optimum. Specifically, using the physiological optimum, slower‐growing *Salvelinus namaycush* maximum attainable lifetime weight was underestimated, and consumption overestimated, while fast‐growing *Oncorhynchus tshawytscha* maximum attainable weight was overestimated. While the physiological optimum approach is useful for theoretical studies, our results demonstrate the critical importance that models used by management utilize up‐to‐date system‐ and species‐specific field data representing actual in‐situ behaviors (i.e., realized thermal niche).

## Graphical abstract

Bioenergetics models are routinely used to estimate food consumption or growth and are thus critical tools in applied science and decision‐making in aquatic ecosystem and species management. Even though fish telemetry is widely used and has enabled researchers to accurately document in‐situ temperatures aligned with dynamic fish behavior, that is, their realized thermal niche, the traditional use of lab‐derived optimum temperatures based on the physiological optimum theory in models still persists. In this study, we contrasted optimum and field temperature data in models and quantified the effects on growth and consumption of two widely distributed salmonids that occupy both marine and freshwater ecosystems. We demonstrate that significant biases in the model estimates of growth and lifetime prey consumption produced using lab‐obtained optimum temperatures could be eliminated by the adoption of realized thermal niche approach to bioenergetics modeling.

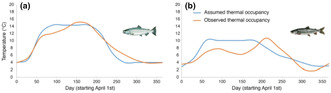

## 1 INTRODUCTION

In an era of global resource management challenges, including climate‐associated changes and extremes, accurate information is crucial for sound and sustainable decision‐making, particularly for fisheries that are critical for feeding the planet (Costello et al., 2020). Fisheries managers rely on growth and consumption estimates from bioenergetics models to inform decisions (e.g., fishery yields) that can affect an ecosystem's health, economy, and services for decades (Chipps & Wahl, 2008; Hansen et al., 1993). While bioenergetics model estimates provide a sound approach to understanding predation pressure and ecosystem productivity for decision‐making purposes, models are only as good as the data used to populate them (Bartell et al., 1986). Uncertainty in data inputs and parameters remains a significant concern because it tends to magnify through the process, from creating a model through to resulting prediction or estimation (Chipps & Wahl, 2008; Hansen et al., 1993). Temperature is an ecological master factor, affecting virtually all biochemical, physiological, and life history processes (Beitinger et al., 2000; Brett, 1971; Fry, 1971). All fish bioenergetics models, including the commonly used Wisconsin model (Deslauriers et al., 2017; Hewett & Johnson, 1987), incorporate temperature‐dependent functions to describe metabolism, consumption and growth. Fish thermal preferences are species‐specific and considering the wide variation in temperatures available in temperate ecosystems, any uncertainty or generalization of the inputs of this factor would have a significant impact on model results and could compromise the effectiveness of any management decisions based on them (Chipps & Wahl, 2008; Deslauriers et al., 2017).

**FIGURE 1:**
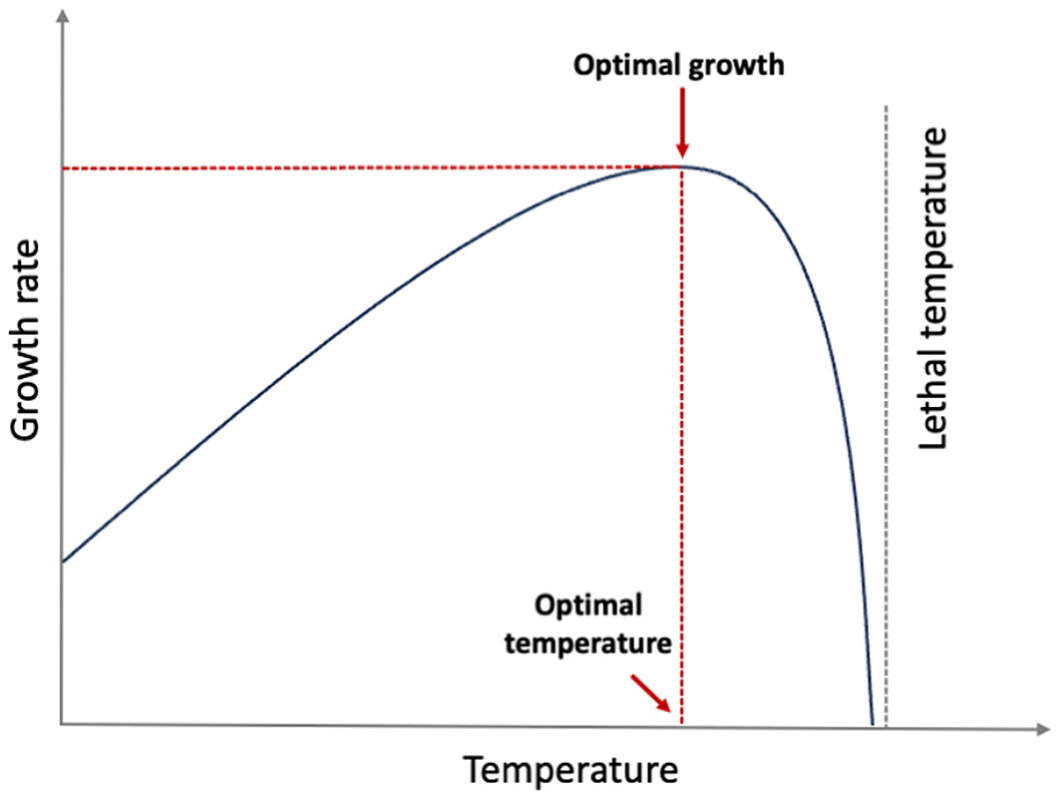
Generalized optimal growth curve for fish.

Within the realized niche of animals, there is a narrow band of optimum conditions where fitness is greatest (Elton, 1927; Tilman, 1980) (Figure 1). The *final preferendum* paradigm states that thermal preference is equal to the final temperature an ectotherm selects when given a temperature gradient (Fry, 1947; Reynolds & Casterlin, 1979). Since physiological consequences of high‐temperature exposure are more extreme, the general premise of bioenergetics is that animals will occupy the temperatures closest to their physiological optimum to optimize growth and survival (Hanson et al., 1997; Stewart, Weininger, et al., 1983). The optimum temperature is widely used in bioenergetics models for generating growth and consumption estimates and decades of research have been devoted to identifying the physiological optimum for different fish species via the final preferendum concept. Those studies were and continue to be conducted under strictly controlled lab conditions, where fish are fed to satiation and there are no predators or competitors (Brett & Groves, 1979; Ney, 1993).

Environmental conditions, however, including abiotic and biotic factors, redefine the optimum conditions for maximizing fitness to include optimizing survival and foraging within the realized niche (Brett et al., 1969; Elton, 1927; Reynolds & Casterlin, 1979; Tilman, 1980). Thus, while the physiological optimum occurs within the realized niche it may differ from the final preferendum when in‐situ. For example, optimum temperatures are sensitive to ration (Huey & Kingsolver, 2019) and reduced food supply has been linked to preference for cooler conditions for common minnow (*Phoxinus phoxinus*), bluegill sunfish (*Lepomis macrochirus*) and coho salmon (*Oncorhynchus kisutch*) (Brett et al., 1969; Killen, 2014; Wildhaber & Crowder, 1990), or to foraging in warmer than the optimum thermal conditions for lake trout (*Salvelinus namaycush*) (Morbey et al., 2006). Other factors that influence the realized niche and the final preferendum include heterogeneity in traits or local thermal adaptations (Angilletta et al., 2004; Englund et al., 2011). Regardless of the underlying reasons, physiological optimum temperature for fish based on the optimum growth curve (as shown in Figure 1) obtained from lab studies may not reflect the actual temperatures occupied within and across years in natural settings (i.e., the realized niche). While the niche concept and the final preferendum paradigm have existed for decades (see Fry, 1947; Grinnell, 1917), the application of the realized niche (i.e., the use of occupied temperatures in the wild) in bioenergetics modeling is still lagging (see below) and the use of the physiological optimum remains a source of uncertainty because it overlooks important behavioral considerations associated with habitat selection.

The implications of species occupying warmer or colder temperatures than their optimum may be significant for bioenergetic predictions and ultimately management. As fish behaviorally thermoregulate, especially under conditions of limited resources (Brett et al., 1969), consumption estimates, for example, would be inflated for fish occupying cooler temperatures than their predicted optimum. Indeed, model consumption estimates for fish have poorer accuracy for cooler compared to warmer temperatures and seasons (e.g., Chipps et al., 2000; Hartman & Brandt, 1993). The development of tracking technologies for aquatic organisms over the last three decades, including acoustic telemetry and archival tags, are contributing to increased knowledge of the behavior and habitat selection of fish (Hussey et al., 2015), and thus, also provide an opportunity for better understanding of their final thermal preferenda and behavioral thermoregulation in the wild. For example, summer temperatures are assumed to be most important for fish growth (Railsback, 2022), yet telemetry studies have demonstrated that non‐summer temperatures have stronger effects on some species (Armstrong et al., 2021; Railsback & Rose, 1999). Importantly, tracking technologies have enabled scientists to build long‐term, year‐round datasets of temperatures occupied by fish in the wild that can be used to inform bioenergetics models, and specifically to identify potential types and levels of bias in growth and consumption estimates.

Bias in bioenergetics model results is a well‐known issue (see Chipps & Wahl, 2008; Ney, 1993) and the assumption of thermal habitat selection near the optimum has been debated since the early onset of those models (e.g., Bevelheimer, 1990; Spigarelli et al., 1982). Lab studies comparing growth and consumption rates at various holding temperatures have shown wide variation in results (e.g., Canale, 2014; Johnson et al., 2023). Comparisons quantifying bias of lab‐obtained thermal versus field‐observed preference of wild populations are rare and indirect (e.g., Brodie et al., 2016) and to our knowledge, none exist that include multiple age classes across all seasons. Considering the exponential increase of animal tracking studies and the availability of in‐situ occupied year‐round temperatures for many fish at various life stages (Hussey et al., 2015; Matley et al., 2021), the use of this data in the field of bioenergetics modeling is lagging. A literature search revealed that during the last decade, of 380 articles using bioenergetics modeling only five used empirically derived field temperatures (from telemetry or archival data), and the rest make an assumption or inference (see Appendix S1). A few studies incorporated limited field observational data (e.g., Bart et al., 2021) and although this strategy may reduce bias, field data that is incomplete and/or inferred is still subject to similar assumptions as those for physiological optimum temperatures. Given this and the lag in use of available field temperature data in fish bioenergetics models, a direct assessment of the influence of assumed/inferred temperatures on model predictions is necessary.

Bioenergetic model predictions help guide management actions (Hansen et al., 1993), yet uncertainties and/or assumptions with input data, such as temperature, compromise their accuracy and utility (Chipps & Wahl, 2008; Ney, 1993). Thus, the objective of this study was to compare and quantify the influence of using the physiological thermal optimum (hereafter “assumed thermal occupancy”) versus the realized thermal niche/preferendum (hereafter “observed thermal occupancy”) on bioenergetics model predictions for two fish with different life history strategies in a large lake. Lake trout and Chinook salmon (*Oncorhynchus tshawytscha*) were chosen as the model species because they have global distribution occupying both freshwater and marine environments (Nelson, 2006), are important recreational, commercial, and subsistence species, and bioenergetic models are widely used for their management (e.g., Deslauriers et al., 2017; Hansen et al., 1993; Ney, 1993). Multi‐year datasets of weight‐at‐age from fishery surveys for each species provided accurate estimates of fish growth for inclusion in the bioenergetics models. Year‐round, multi‐year, in‐situ temperature datasets obtained using acoustic telemetry and archival tags (Ivanova, Larocque, Fisk, & Johnson, 2021; Raby et al., 2017) provided the observed thermal occupancy for each species, with the dataset for Chinook salmon spanning juvenile to adult age‐classes.

## 2 MATERIALS AND METHODS

### 2.1 Study system

Lake Ontario (43.6333° N, 77.8271° W) is part of the Laurentian Great Lakes in North America. Its surface area extends 19,000 km^2^ and its depth reaches 245 m, making it the 10th largest freshwater lake globally by volume. Lake Ontario is dimictic and experiences a wide range of temperatures (0 to >20°C) above 20 m depth across seasons but year‐round colder temperatures (<4°C) at depth (Ivanova, Johnson, Metcalfe, & Fisk, 2021). It is a highly productive ecosystem containing >100 fish species, including six salmonids (Mills et al., 2003). Due to the importance for the US and Canadian economies, the Lake Ontario ecosystem and its fish populations are highly managed.

### 2.2 Study species

Both, lake trout and Chinook salmon are top predators in the Great Lakes and Lake Ontario reaching a length of 1 m at adulthood. Both species have cold‐water preference but different lab‐derived optimum temperatures (10°C for lake trout [Stewart, Weininger, et al., 1983] and 14.3°C for Chinook salmon [Hasnain et al., 2010]) and although both grow to similar sizes they have different life histories affecting growth rate; lake trout is iteroparous with a lifespan of 25+ years, while Chinook salmon is semelparous and lives 3–4 years (Jacobs et al., 2013; Mumby et al., 2018; Quinn, 2005; Stewart, Kitchell, & Crowder, 1983). Additionally, lake trout is a diet generalist, while Chinook salmon is considered a diet specialist in the Great Lakes (Jacobs et al., 2013; Mumby et al., 2018; Quinn, 2005; Stewart, Kitchell, & Crowder, 1983).

### 2.3 Telemetry

All methods for the acoustic telemetry and pop‐off data storage tags (pDST), including the ethical board approvals, have been provided in detail in Ivanova et al. (2022), Ivanova, Johnson, Metcalfe, and Fisk (2021) and Raby et al. (2017), respectively. Briefly, 278 permanent acoustic telemetry receivers (69 kHz, VR2W, Innovasea Systems Inc., Bedford, Nova Scotia, Canada) spaced between 2 and 15 km apart were used to detect fish tagged with acoustic transmitters in Lake Ontario (Appendix S2: Figure S1). Juvenile Chinook salmon (*n* = 45) were tagged at both the eastern and western ends of the lake in 2017–2018 with V13 depth and temperature sensor tags (45 mm length × 13 mm diameter; 6 g weight in water; nominal delay 180 s; estimated battery life 703 days; Innovasea Systems Inc.). Lake trout (*n* = 42) were tagged only at the eastern end of the lake during the same period with V16 depth and temperature sensor tags (68 mm length × 16 mm diameter; 10.3 g weight in water; nominal delay 180 s; estimated battery life 3650 days; Innovasea Systems Inc.). Lake trout were gill‐netted and Chinook salmon were angled and kept in a 50 L tank filled with lake water until surgery. Surgeries lasted <3 min and involved an incision anterior of the pelvic fins, implantation of the tag in the body cavity, three interrupted sutures to close the incision and tagging with an external floy tag. For pDST tagging, all fish (*n* = 32 Chinook salmon and *n* = 40 lake trout) were angled in 2014 to 2016 and an external harness was attached through the dorsal musculature. The pDST tags (a time‐release G5 long‐life 20 bar depth‐temperature logger with a float; Cefas Technology Inc.) were programmed to log temperature (precision 0.03125°C and accuracy ±0.1°C) every 70 s. Fish were allowed to recover in an aerated tank until upright swimming was restored prior to release for both types of tagging. A total of 10 Chinook salmon and 18 lake trout with acoustic telemetry tags and 11 of each species with pDST tags were included in the calculations of present temperatures.

### 2.4 Water temperature scenarios

Two temperature scenarios were used to understand the importance of observed thermal occupancy on estimates of growth and consumption: (i) the assumed thermal occupancy scenario (or “assumed occupancy” for short), based on the physiological optimum temperature for growth (optimal growth theory), used lab derived optimum temperature preferences for the two model species; and, (ii) the observed thermal occupancy scenario (“observed occupancy”), representing the realized final thermal preferendum, used temperatures collected in‐situ for the two model species between 2014 and 2020.

**FIGURE 2:**
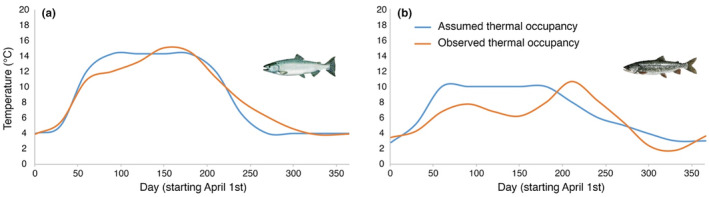
Distribution of temperatures under assumed thermal occupancy and observed thermal occupancy (realized thermal preferendum) scenarios for (a) Chinook salmon and (b) lake trout. *Note*: *Assumed thermal occupancy* represents the lab‐obtained optimum temperature for the species and occupancy of the warmest temperature available in the habitat up to but not exceeding the optimum temperature. *Observed thermal occupancy* represents the observed temperatures occupied by the species in Lake Ontario between 2014 and 2020.

Assumed optimum thermal occupancies for lake trout or Chinook salmon in Lake Ontario were 10°C (Stewart, Weininger, et al., 1983) and 14.3°C (Hasnain et al., 2010), respectively, and if this temperature was not available in the ecosystem due to seasonality, the warmest temperature available not exceeding these optima (as per Stewart, Weininger, et al., 1983) was used based on temperatures observed in, Ivanova, Johnson, Metcalfe, and Fisk (2021), (Figure 2). Warmest temperatures across the lake were obtained from satellite‐derived lake surface temperatures available through NOAA GLERL CoastWatch Program (https://coastwatch.glerl.noaa.gov/satellite‐data‐products/great‐lakes‐surface‐environmental‐analysis‐glsea).

Observed temperatures occupied by individuals were obtained from acoustic telemetry temperature sensor tags and pDST tags. Daily mean temperature used by each species was calculated from the two datasets. From those daily values, an average temperature was calculated for every 30th day starting April 1 for lake trout and September 1 for Chinook salmon based on a 10‐day mean before and after the 30‐day mark. Modeling was done in Fish Bioenergetics 4.0, where, commonly, for multi‐year analyses of fish growth rate potential, temperature at 30‐day intervals throughout the year is used and the software interpolates the values between these days for smoothing purposes (Deslauriers et al., 2017). Even though daily mean temperature was available for the empirical data, this 30‐day interval was used to avoid bias. September 1 for Chinook salmon was chosen because juveniles would have descended from the tributaries and have become lake residents by this date, and because all data for weight‐at‐age was collected in August and September (2010–2019; source: U.S. Geological Survey). The resulting temperatures were used as input values in the bioenergetics models and were presumed to remain steady throughout the lifetime of the modeled fish.

### 2.5 Predator energy density, equations, and modeling simulations

To estimate energy density of lake trout in Lake Ontario, whole‐body lipid data (source: Environment and Climate Change Canada) from 1977 to 2019 (*n* = 5766; Appendix S2: Table S1) were used with a conversion of 39.5 kJ/g (Brody, 1945). These values were then compared to actual energy density values obtained by bomb calorimetry of whole animals (*n* = 22; source: Ontario Ministry of Natural Resources and Forestry) and the difference of the means between the two methods was converted to a percentage and used to scale the lipid energy density values. Calorimetry values averaged 12.1% higher than lipid‐based estimates and an adjustment of the lipid‐based estimates was made.

For calculating the Lake Ontario‐specific energy density equation, weights >8000 g were infrequently reported and were therefore removed from further analysis. Weight dependence of energy density was established using the piecewise.linear() function from the SiZer package (Sonderegger et al., 2009) in R with 1000 iterations and a significance level of 0.001 to estimate the break‐point for separating immatures from adults and fit the two linear models. Based on the estimated breakpoint, the two linear models were fitted, and the equations of the lines were used in the simulations.

Chinook salmon skinless, boneless filet lipid data (source: Ontario Ministry of the Environment, Conservation and Parks) from 1976 to 2018 (*n* = 1390) were used to calculate energy density based on the following lipid‐energy density equation by Trudel et al. (2005):

$$\text{Energy density}\;\left(\mathrm{kJ}/g\;W\right)=3.60+0.047^{*}\;\text{Whole}-\text{body lipids}\;\left(\mathrm{mg}/g\;W\right).$$

Considering the above equation is for whole body lipids, the Chinook energy density values were compared to those of coho salmon for skinless, boneless filet, and whole‐body. Since the filet energy density values between Chinook and coho were not significantly different (two‐sided Student's *t*‐test, *p* = .25), the difference of the means between the coho filet and whole‐body energy density values was converted to percentage and used to scale the Chinook energy density values. Coho whole‐body calorimetry values averaged 34.6% higher than the lipid‐based estimates, thus the latter for Chinook were adjusted accordingly.

For calculating the Lake Ontario‐specific energy density equation for Chinook salmon, values for the regression were limited to fish of sizes 200–15,000 g (Appendix S2: Table S1). The piecewise.linear() function did not yield a meaningful break‐point for separating juveniles' from adults' energy density equations, so one equation was used for all ages for further analysis.

We used the Wisconsin bioenergetics model to simulate growth (Deslauriers et al., 2017; Kitchell et al., 1977). For each species, we replaced the energy density equations with those calculated from the available data for Lake Ontario. All other physiological parameters were kept as set in the software. Model sensitivity and assumptions are summarized in references (Bartell et al., 1986; Madenjian et al., 2000; Ney, 1990, 1993). Simulations start day for lake trout was April 1 and for Chinook salmon September 1.

To assess the lifetime growth rate potential and consumption of the species under each scenario, lifetime growth was simulated for an individual's lifespan from age 1 through 15 for lake trout and 0 through 4 for Chinook salmon. Lake trout starting weight at age class 1 was 142 g and Chinook salmon at age class 0 was 200 g. Feeding rate (also known as *p*‐value and defined as proportion of the maximum consumption [Beauchamp et al., 2007]) and consumption were calculated from the observed temperatures for the same scenario using the fit‐to‐weight function in the modeling software. Using the fit‐to‐feeding rate function and the assumed optimum temperatures, we calculated the final weight at adulthood and lifetime consumption for the assumed occupancy scenario.

Age‐specific consumption rates (in g of prey per g of predator per day, i.e., g/g/d) were calculated in the same manner as described in the above paragraph for adult lake trout age 14 and Chinook salmon age 4. In both thermal occupancy scenarios, starting and final weights‐at‐age were 6573 and 6980 g for lake trout (ages 13 and 14) and 9742 and 10,387 g for Chinook salmon (ages 3–4), respectively.

### 2.6 Prey energy density, diet composition, and digestibility

Prey energy density was obtained from the literature (for details and associated references see Appendix S2: Table S2) and where multiple values for a species were available, a mean was calculated. Prey diet composition and proportions for the two predators and associated references are provided at https://github.com/ivanovas‐g/diet_change_proportions_LT‐CS.git. Prey digestibility was assumed to be the same across all prey species for both predators. Indigestible prey proportion used was 0.033 (Stewart, Weininger, et al., 1983).

## 3 RESULTS

### 3.1 Predator energy density

For improved model accuracy, energy density equations for each species were developed from Lake Ontario‐specific data. Lake trout exhibited a nonlinear relationship between energy density and weight with a transition between juveniles (individuals that have not reached sexual maturity and/or are still experiencing greater relative growth) and adults occurring at 1627 g (*p* < .001, residual error of 1.762 and DF 5762; Appendix S2: Figure S2), necessitating separate equations:

(1) $$\text{Juvenile}:y=2.21+0.00328^{*}\;\text{Weight}$$

and,

(2) $$\text{Adult}:y=6.74+0.000501^{*}\;\text{Weight}.$$

There was no distinction between juvenile and adult Chinook salmon energy density and weight relationship, thus one equation was used for all ages (Appendix S2: Figure S2):

(3) $$y=6.11+0.0000983^{*}\;\text{Weight}.$$

### 3.2 Thermal occupancy

Observed thermal occupancy based on acoustic telemetry and pDST tags was different from the assumed optimal thermal occupancy for both species. Tag data showed that Chinook salmon occupied cooler temperatures in the spring but warmer in mid‐summer and during the fall compared to the assumed thermal optimum (Figure 2a). Lake trout were found at lower temperatures than their assumed thermal optimum for all months except April, October, and November (Figure 2b).

### 3.3 Modeled growth and consumption

**FIGURE 3:**
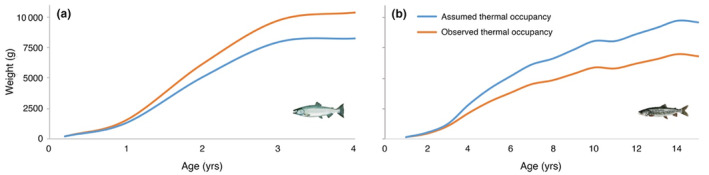
Lifetime growth response to temperature. Weight (in g) achieved per age for Chinook salmon (a) and lake trout (b) under assumed (blue) and observed (orange) thermal occupancy scenarios in Lake Ontario. *Note*: observed thermal occupancy weight is based on weight‐at‐age data obtained from 2010 to 2019.

**FIGURE 4:**
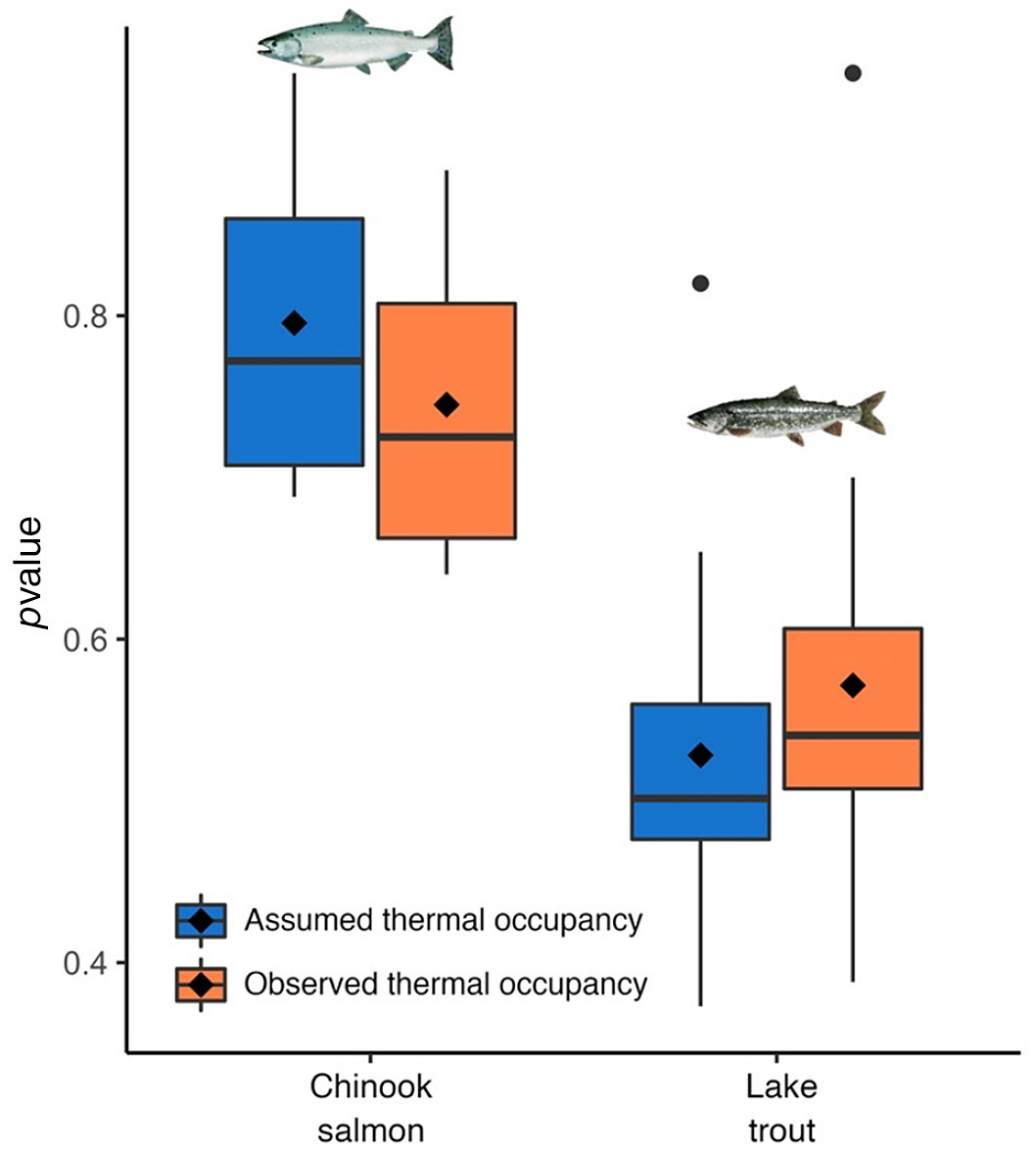
Lifetime *p*value (feeding rate) distributions. *p*value (i.e., defined as proportion of the maximum consumption) for Chinook salmon and lake trout with assumed (blue) and observed (orange) thermal occupancy in Lake Ontario. For both species, the *p*values were statistically different between thermal occupancies (*p* < .001, Student's paired *t*‐test). Mean is shown with diamond, median with a thick black line and whiskers represent the data distribution.

Chinook salmon weight‐at‐age 4 (maximum age) based on lifespan calculations was underestimated by 20.5% (Figure 3a) under assumed thermal occupancy, whereas the weight‐at‐age 15 for lake trout was overestimated by 29.2% (Figure 3b). Feeding rate‐at‐age was significantly different between assumed and observed thermal occupancy for each species (*p* < .001, Student's paired *t*‐test; Figure 4), lake trout had values of 0.53 ± 0.11 (mean ± 1 SD) and 0.57 ± 0.14, respectively, and Chinook salmon 0.8 ± 0.12 and 0.75 ± 0.11, respectively.

**FIGURE 5:**
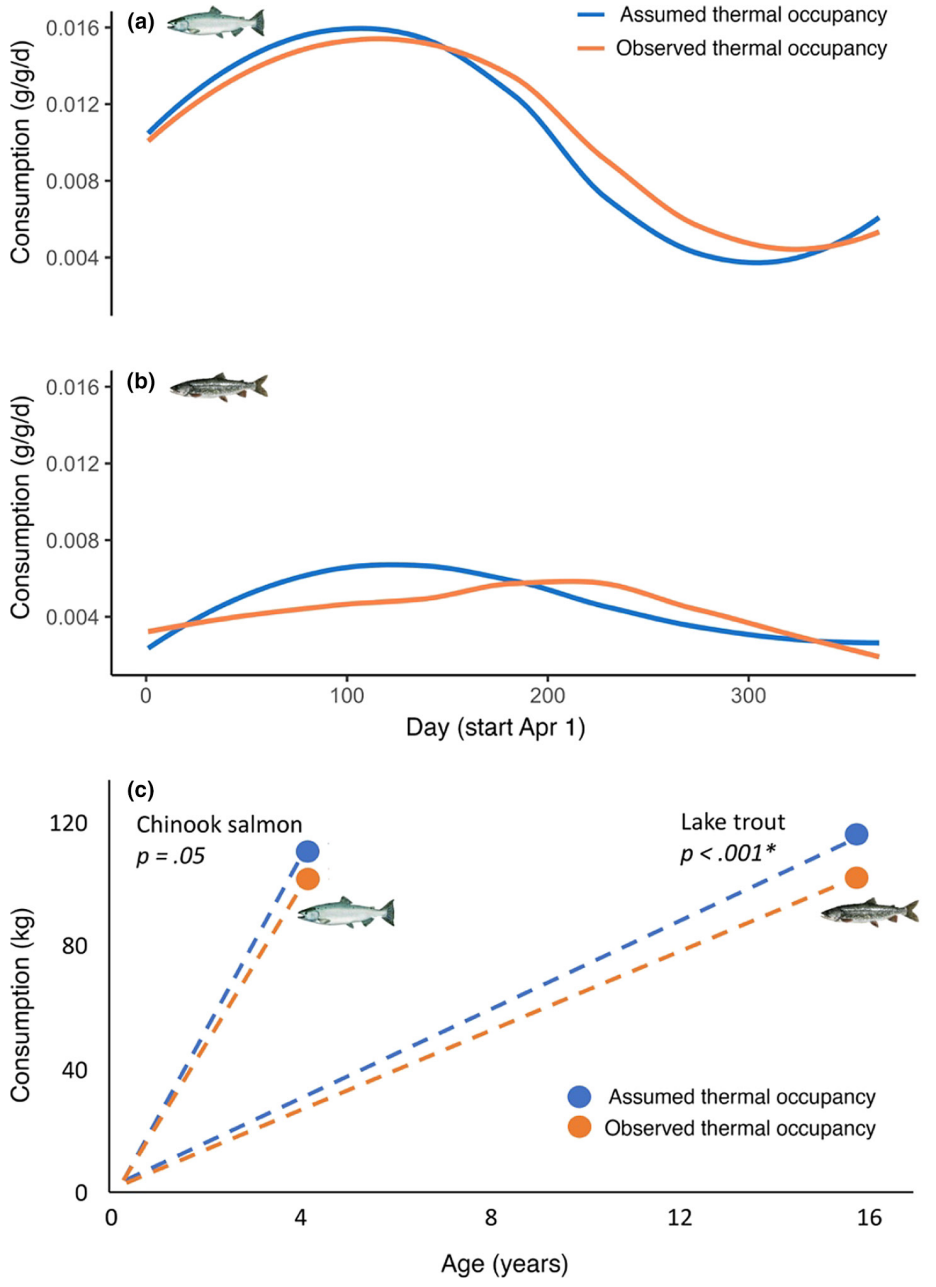
Age‐specific daily and total lifetime consumption estimates. Estimated daily age‐specific consumption (a and b) was significantly different (*p* < .001, Student's paired *t*‐test for both species) between assumed optimum and observed thermal occupancy models for adult (a) Chinook Salmon age 4 and (b) lake trout age 14. Model‐estimated total lifetime consumption between assumed and observed thermal occupancy (c) was significantly different for lake trout, but not for Chinook salmon.

Daily age‐specific consumption for adult Chinook salmon (i.e., age 4; Figure 5a) and lake trout age (i.e., age 14; Figure 5b) estimated from the bioenergetic models was significantly different (*p* < .001, Student's paired t‐test for both species) between assumed and observed thermal occupancy scenarios. Across the entire year, the daily age‐specific consumption for age‐4 Chinook salmon based on assumed thermal occupancy was 3.1% lower than observed occupancy, and for age‐14 lake trout was 7.1% higher. Total lifetime consumption between assumed and observed thermal occupancy was significantly different for lake trout (*p* < .001) but not significantly different (*p* = .05) for Chinook salmon (Figure 5c; Student's paired *t*‐test for both species). For a single individual fish, the total lifetime prey consumption for assumed and observed thermal occupancy for Chinook salmon was 114 and 109 kg, respectively, and for lake trout was 119 and 110 kg, respectively.

## 4 DISCUSSION

Accurate input data that reduces uncertainties and minimizes model bias are critical when developing predictions to support management decisions that affect ecosystems and the communities dependent on them. Fish growth and consumption are important metrics for species and ecosystem management (Deslauriers et al., 2017; Ney, 1993) and are estimated using bioenergetics models. Yet, existing bioenergetics models for ectotherms employ an assumed thermal occupancy (i.e., physiological optimum) approach that is based on controlled laboratory studies (where animals are fed to satiation) and assumed maximum growth (Brandt et al., 2002; Stewart, Weininger, et al., 1983). This approach ignores the niche theory and its spatial realization because organismal temperature preference in the wild is a function of environmental abiotic and biotic conditions (e.g., prey availability and density, conspecific density) and behavior (e.g., interactions with other species) (Huntsman et al., 2021; Killen, 2014; Wildhaber & Crowder, 1990). Here, we tested this inference using in‐situ observed thermal occupancy data for two fish species and demonstrated that assumed thermal occupancy in bioenergetic modeling can under‐ or over‐estimate growth and consumption depending on the species. Our study shows how temperature alone can produce a significant bias in model estimates, demonstrating that the realized niche, that is, observed thermal occupancy, is a more robust approach for bioenergetics modeling. Further, the increased use of logger technologies worldwide provides an efficient way to describe realized habitat occupancy, thus minimizing temperature bias. This is particularly relevant for management given the continuing and accelerating temperature changes across the globe in aquatic ecosystems (Woolway et al., 2020).

Any overestimation of occupied temperature will quickly inflate predictions of energetic demand during modeling, and here differences between observed and assumed temperatures were greatest for both species in the summer. In our study, observed temperatures for lake trout were lower than assumed for ~9.5 months, and were higher only when the species were moving to shallower areas to spawn in the early fall (Fitzsimons, 1995) or in the spring as they move offshore to deeper waters (Ivanova, Johnson, & Fisk, 2021; Ivanova, Johnson, Metcalfe, & Fisk, 2021). This pattern is consistent with observations from smaller lakes (Olson et al., 1988; Plumb et al., 2014) and for Lakes Michigan and Huron (Bergstedt et al., 2012). For Chinook salmon, the observed occupancy was much more comparable to the optimum in this and other Great Lakes (Olson et al., 1988), but a lower autumnal thermal niche was reported for Chinook salmon in native coastal marine habitats in Oregon and California (Hinke et al., 2005). Given the relative influence of warmer temperatures in inflating bioenergetics estimates, several studies have combined observed summer temperatures with assumed temperatures for other times of the year (Bart et al., 2021; Olson et al., 1988; Stewart & Ibarra, 1991) and our analyses and results suggest these studies also have predictive bias.

In our study, physiology and longevity were key factors that drove differences in bioenergetics modeling results for the two species, consistent with the dynamic energy budget theory (Kooijman, 2000; Ross & Nisbet, 1990). For comparable size and temperature, metabolic costs are 13% higher for Chinook salmon than lake trout and differences increased with rising temperature (Hanson et al., 1997; Stewart & Ibarra, 1991). Higher metabolic costs necessitate more or higher quality food to attain comparable growth. Likewise, Chinook are much shorter lived than lake trout, and thus food consumption rates must be substantially greater to support the larger annual growth increment as both fish can attain similar maximum adult weight. Some bioenergetics models rely on data and parameters borrowed from other species, and previous studies have emphasized the importance of understanding the implications of such actions on model accuracy (Chipps & Wahl, 2008; Deslauriers et al., 2017; Ney, 1993). As such, we corroborate that physiologic and life history differences need to be considered when developing model predictions.

Habitat selection is influenced by different biotic factors, such as prey distribution and abundance (Grenier‐Potvin et al., 2021; Jepsen et al., 2002). Here, these are possible drivers of the deviations in observed compared to the assumed thermal occupancy for the species in this study. Both of those factors have different impacts on species with different life histories. Considering that organisms try to minimize search time and maximize foraging success (Pyke, 1984), Chinook salmon, which are diet specialist in the Great Lakes (Mumby et al., 2018), have to follow their primary prey alewife (*Alosa pseudoharengus*) to meet their energetic demands – temperatures occupied by Chinook salmon are largely consistent with those reported in the literature for alewife (Holden et al., 2018; Weidel et al., 2018). Lake trout, however, are more generalist in their diet (Colborne et al., 2016; Mumby et al., 2018) and are able to forage opportunistically on different prey in proximity. Given the generally lower than optimum thermal occupancy of lake trout in‐situ (except in the fall during spawning time) based on our field data, either prey are occupying those lower temperatures or overall prey abundance is lower and the species are optimizing growth for lower ration intake (Huey & Kingsolver, 2019). Other factors affecting habitat selection are predation (e.g., Jordan et al., 1997; although not the case for Chinook salmon and lake trout in Lake Ontario as they are the top predators in the system) and competition (Grenier‐Potvin et al., 2021). Lake trout is a weak competitor that would switch prey and foraging areas in the presence of strong competitors (Vander Zanden et al., 1999) such as Chinook salmon. Overall, while understanding the drivers for the deviation of in‐situ occupied temperatures compared to optimal is important, it still remains difficult to predict how different drivers may interact to influence habitat selection and thermoregulation behavior.

In an era of balancing between maximizing fishery yields and threshold harvesting to ensure sustaining fish populations, field data‐driven models with accurate predictions are crucial for effective management (Ney, 1993). Sensitivity analyses of bioenergetic models suggest estimates ±10% are considered *good* (Bartell et al., 1986; Brandt et al., 1992; Madenjian et al., 2000; Ney, 1990, 1993), but this variation may, in reality, be larger due to the combined effect of other parameter and input data uncertainties, such as routine energy expenditure (Barnett et al., 2017). In their review, Chipps and Wahl (2008) showed the majority of model consumption results routinely exceeded 10% with some surpassing 100% (over‐ or underestimation) when compared to both field and lab studies and concluded estimates were particularly positively influenced by temperature and ration levels. Given the above, we echo Ney (1993) call for accurate field data and amend it to recommend that models used for system‐specific applied science should utilize year‐round system‐ and species‐specific field data that represent the realized thermal preferenda of the species to produce more accurate predictions.

Limited resources of agencies and management often restrict the employment and application of new technologies for developed and underdeveloped nations, necessitating an approximation to inform decision‐making. Yet, cases like the collapse of Atlantic cod (*Gadus morhua*) in the early 1990s demonstrated that assumptions and inaccuracies can have detrimental consequences to populations (Hutchings & Myers, 1994). One way to overcome resource limitations is through extensive partnerships with academia and other agencies to either combine funding and projects, pursue similar projects, or extend the use of data that is already being collected for other studies (Krueger et al., 2018). Alternatively, if reliance on inference or optimal temperature is still the only option until new data is available, we propose that decision‐makers remain conservative in their approach to setting quotas.

Ecosystem management relies on model estimates to make decisions concerning the rapidly changing environmental conditions that challenge fish fitness and survival. While evidence shows that climate change impacts would be mediated by a species' life history and thermal preferences (i.e., cold vs. warm) (Alofs et al., 2014; Collingsworth et al., 2017; Lynch et al., 2016), predicting some of these effects and their severity may be encumbered by assumptions, such as the use of assumed temperatures in models. For example, Kraus et al. (2015) pointed out that the thermal niche for striped bass (*Morone saxatilis*) needs to be revised to include warmer temperatures based on observations in the Patuxent River. An increase in estimate and prediction accuracy of models would improve substantially our understanding of the types and intensity of impacts the changing climate has on a species, the community it occupies, and the entire ecosystem.

## 5 CONCLUSION

Management application of bioenergetic models varies from estimates of predator–prey interactions dynamics to productivity and nutrient cycling within aquatic food webs to food consumption of animals, their populations, and communities. While parameter and input data uncertainties in models are a known problem, direct quantification of bias is often difficult to obtain and, thus, bias is difficult to address. Based on direct comparisons, our study provides quantitative evidence that temperature alone, in this case the use of assumed thermal occupancy (i.e., lab‐obtained physiological optimal temperature) based on the optimal growth theory, produces significant bias on bioenergetics model results. More importantly, we demonstrate that the bias produced by temperature can be reduced significantly if the realized thermal niche concept is applied because it removes the need to make assumptions regarding habitat selection. The assumed optimum occupancy may still be useful for bioenergetics modeling where the goal is to increase understanding at continental or global scales or overall theoretical knowledge. However, at local ecosystem scales where predictions are used for informing decisions, management should be vigilant that the models utilize in‐situ collected continuous year‐round species‐ and system‐specific data to eliminate behavioral or niche bias.

## AUTHOR CONTRIBUTIONS

**Silviya V. Ivanova:** Conceptualization (lead); formal analysis (lead); investigation (lead); methodology (lead); visualization (lead); writing – original draft (lead); writing – review and editing (equal). **Aaron T. Fisk:** Conceptualization (supporting); funding acquisition (lead); investigation (supporting); project administration (equal); supervision (equal); writing – review and editing (equal). **Timothy B. Johnson:** Conceptualization (equal); formal analysis (supporting); funding acquisition (equal); investigation (equal); methodology (equal); project administration (equal); supervision (equal); writing – review and editing (equal).

## CONFLICT OF INTEREST STATEMENT

The authors declare no conflicts of interests.

## STATEMENT ON INCLUSION

Our study brings together authors based in the country where the study was carried out. All authors were engaged early on with the research and study design to ensure that the diverse sets of perspectives they represent (i.e., both academia/theorists and government/management) were considered from the onset. Whenever relevant, literature published by scientists from the region was cited.

## Supporting information


Appendix S1‐S2

## Data Availability

Custom parameter data have been provided via the following link: https://github.com/ivanovas‐g/diet_change_proportions_LT‐CS.git. Storage tag data are publicly archived at the Dryad Digital Repository https://doi.org/10.5061/dryad.kn02d60. Acoustic telemetry temperature data are publicly archived at the *Zenodo* repository https://doi.org/10.5281/zenodo10257697. *All code used for analysis is standard and cited as necessary*.

## References

[ece310974-bib-0001] Alofs, K. M. , Jackson, D. A. , & Lester, N. P. (2014). Ontario freshwater fishes demonstrate differing range‐boundary shifts in a warming climate. Diversity and Distributions, 20(2), 123–136. 10.1111/ddi.12130

[ece310974-bib-0002] Angilletta, M. J. , Steury, T. D. , & Sears, M. W. (2004). Temperature, growth rate, and body size in ectotherms: Fitting pieces of a life‐history puzzle. Integrative and Comparative Biology, 44(6), 498–509. 10.1093/icb/44.6.498 21676736

[ece310974-bib-0003] Armstrong, J. B. , Fullerton, A. H. , Jordan, C. E. , Ebersole, J. L. , Ryan Bellmore, J. , Arismendi, I. , Penaluna, B. E. , & Reeves, G. H. (2021). The importance of warm habitat to the growth regime of cold‐water fishes. Nature Climate Change, 11(4), 354–361. 10.1038/s41558-021-00994-y PMC9037341 35475125

[ece310974-bib-0004] Barnett, A. , Braccini, M. , Dudgeon, C. L. , Payne, N. L. , Abrantes, K. G. , Sheaves, M. , & Snelling, E. P. (2017). The utility of bioenergetics modelling in quantifying predation rates of marine apex predators: Ecological and fisheries implications. Scientific Reports, 7(1), 12982. 10.1038/s41598-017-13388-y 29021551 PMC5636836

[ece310974-bib-0005] Bart, R. J. , DeVries, D. R. , & Wright, R. A. (2021). Change in piscivore growth potential after the Introduction of a nonnative prey fish: A bioenergetics analysis. Transactions of the American Fisheries Society, 150(2), 175–188. 10.1002/tafs.10276

[ece310974-bib-0006] Bartell, S. M. , Breck, J. E. , Gardner, R. H. , & Brenkert, A. L. (1986). Individual parameter perturbation and error analysis of fish bioenergetics models. Canadian Journal of Fisheries and Aquatic Sciences, 43(1), 160–168. 10.1139/f86-018

[ece310974-bib-0007] Beauchamp, D. A. , Cross, A. D. , Armstrong, J. , Myers, K. W. , Moss, J. H. H. , Boldt, J. , & Haldorson, L. J. (2007). Bioenergetic responses by Pacific Salmon to climate and ecosystem variation. North Pacific Anadromous Fish Commission, 4(4), 257–269.

[ece310974-bib-0008] Beitinger, T. L. , Bennett, W. A. , & McCauley, R. W. (2000). Temperature tolerances of north American freshwater fishes exposed to dynamic changes in temperature. Environmental Biology of Fishes, 58(3), 237–275. 10.1023/A:1007676325825

[ece310974-bib-0009] Bergstedt, R. A. , Argyle, R. L. , Krueger, C. C. , & Taylor, W. W. (2012). Bathythermal habitat use by strains of great lakes‐and finger lakes‐origin lake trout in lake Huron after a change in prey fish abundance and composition. Transactions of the American Fisheries Society, 141(2), 263–274. 10.1080/00028487.2011.651069

[ece310974-bib-0010] Bevelheimer, M. S. (1990). *Habitat selection by kokanee Salmon and Smallmouth bass in thermally hetero‐ Geneous environments: The importance of growth Maximizationtodiel habitat shifts*. Doctoral dissertation. University of Tennessee, Knoxville.

[ece310974-bib-0011] Brandt, S. B. , Mason, D. M. , McCormick, M. J. , Lofgren, B. , Hunter, T. S. , & Tyler, J. A. (2002). Climate change: Implications for fish growth performance in the Great Lakes. American Fisheries Society Symposium, 2002(32), 61–76.

[ece310974-bib-0012] Brandt, S. B. , Mason, D. M. , & Vincent Patrick, E. (1992). Spatially‐explicit models of fish growth rate. Fisheries, 17(2), 23–35. 10.1577/1548-8446(1992)017<0023:SMOFGR>2.0.CO;2

[ece310974-bib-0013] Brett, J. R. (1971). Energetic responses of Salmon to temperature. A study of some thermal relations in the physiology and freshwater ecology of sockeye Salmon (*Oncorhynchus nerka*). American Zoologist, 11(1), 99–113. 10.1093/icb/11.1.99

[ece310974-bib-0014] Brett, J. R. , & Groves, T. D. D. (1979). Physiological energetics. Fish Physiology, 8(C), 279–352. 10.1016/S1546-5098(08)60029-1

[ece310974-bib-0015] Brett, J. R. , Shelbourn, J. E. , & Shoop, C. T. (1969). Growth rate and body composition of fingerling sockeye salmon, *Oncorhynchus nerka*, in relation to temperature and ration size. Journal of the Fisheries Research Board of Canada, 26(9), 2363–2394. 10.1139/f69-230

[ece310974-bib-0016] Brodie, S. , Taylor, M. D. , Smith, J. A. , Suthers, I. M. , Gray, C. A. , & Payne, N. L. (2016). Improving consumption rate estimates by incorporating wild activity into a bioenergetics model. Ecology and Evolution, 6(8), 2262–2274. 10.1002/ece3.2027 27069576 PMC4782250

[ece310974-bib-0017] Brody, S. (1945). Bioenergetics and growth. Reinhold Publishing Corp.

[ece310974-bib-0018] Canale, R. P. (2014). Modeling juvenile salmonid hatchery growth using a local equilibrium assumption and measured water fraction to parameterize fish energy density. Aquaculture, 434(October), 5–10. 10.1016/j.aquaculture.2014.07.011

[ece310974-bib-0019] Chipps, S. R. , Einfalt, L. M. , & Wahl, D. H. (2000). Growth and food consumption by tiger muskellunge: Effects of temperature and ration level on bioenergetic model predictions. Transactions of the American Fisheries Society, 129(1), 186–193. 10.1577/1548-8659(2000)129<0186:GAFCBT>2.0.CO;2

[ece310974-bib-0020] Chipps, S. R. , & Wahl, D. H. (2008). Bioenergetics modeling in the 21st century: Reviewing new insights and revisiting old constraints. Transactions of the American Fisheries Society, 137(1), 298–313. 10.1577/t05-236.1

[ece310974-bib-0021] Colborne, S. F. , Rush, S. A. , Paterson, G. , Johnson, T. B. , Lantry, B. F. , & Fisk, A. T. (2016). Estimates of Lake trout (*Salvelinus namaycush*) diet in Lake Ontario using two and three isotope mixing models. Journal of Great Lakes Research, 42, 695–702. 10.1016/j.jglr.2016.03.010

[ece310974-bib-0022] Collingsworth, P. D. , Bunnell, D. B. , Murray, M. W. , Kao, Y. C. , Feiner, Z. S. , Claramunt, R. M. , Lofgren, B. M. , Höök, T. O. , & Ludsin, S. A. (2017). Climate change as a long‐term stressor for the fisheries of the Laurentian Great Lakes of North America. Reviews in Fish Biology and Fisheries, 27(2), 363–391. 10.1007/s11160-017-9480-3

[ece310974-bib-0023] Costello, C. , Cao, L. , Gelcich, S. , Cisneros‐Mata, M. Á. , Free, C. M. , Froehlich, H. E. , Golden, C. D. , Ishimura, G. , Maier, J. , Macadam‐Somer, I. , Mangin, T. , Melnychuk, M. C. , Miyahara, M. , de Moor, C. L. , Naylor, R. , Nøstbakken, L. , Ojea, E. , O'Reilly, E. , Parma, A. M. , … Lubchenco, J. (2020). The future of food from the sea. Nature, 588(7836), 95–100. 10.1038/s41586-020-2616-y 32814903

[ece310974-bib-0024] Deslauriers, D. , Chipps, S. R. , Breck, J. E. , Rice, J. A. , & Madenjian, C. P. (2017). Fish bioenergetics 4.0: An R‐based modeling application. Fisheries, 42(11), 586–596. 10.1080/03632415.2017.1377558

[ece310974-bib-0025] Elton, C. (1927). Animal ecology. Edited by J Huxley. Macmillan Co.

[ece310974-bib-0026] Englund, G. , Öhlund, G. , Hein, C. L. , & Diehl, S. (2011). Temperature dependence of the functional response. Ecology Letters, 14(9), 914–921. 10.1111/j.1461-0248.2011.01661.x 21752171

[ece310974-bib-0027] Fitzsimons, J. D. (1995). Assessment of Lake trout spawning habitat and egg deposition and survival in Lake Ontario. Journal of Great Lakes Research, 21(Suppl. 1), 337–347. 10.1016/S0380-1330(95)71108-X

[ece310974-bib-0028] Fry, F. E. J. (1947). Effects of the environment on animal activity. Ontario Fisheries Research Laboratory Publication, Biological Series, 55, 68.

[ece310974-bib-0029] Fry, F. E. J. (1971). The effect of environmental factors on the physiology of fish. In W. S. Hoar & D. J. Randall (Eds.), Fish physiology: Environmental relations and behaviour (pp. 1–98). Academic Press. 10.1016/S1546-5098(08)60146-6

[ece310974-bib-0030] Grenier‐Potvin, A. , Clermont, J. , Gauthier, G. , & Berteaux, D. (2021). Prey and habitat distribution are not enough to explain predator habitat selection: Addressing intraspecific interactions, behavioural state and time. Movement Ecology, 9(1), 12. 10.1186/s40462-021-00250-0 33743833 PMC7981948

[ece310974-bib-0031] Grinnell, J. (1917). Field tests of theories concerning distributional control. The American Naturalist, 51(602), 115–128.

[ece310974-bib-0032] Hansen, M. J. , Boisclair, D. , Brandt, S. B. , Hewett, S. W. , Kitchell, J. F. , Lucas, M. C. , & Ney, J. J. (1993). Applications of bioenergetics models to fish ecology and management: Where do we go from here? Transactions of the American Fisheries Society, 122(5), 1019–1030. 10.1577/1548-8659(1993)122<1019:AOBMTF>2.3.CO;2

[ece310974-bib-0033] Hanson, P. C. , Johnson, T. B. , Schindler, D. E. , & Kitchell, J. F. (1997). *Fish bioenergetics 3.0 software for windows*. University of Wisconsin Center for Limnology, Sea Grant Institute, Technical Report WISCU T‐97‐001, Madison, Wisconsin.

[ece310974-bib-0034] Hartman, K. J. , & Brandt, S. B. (1993). Systematic sources of bias in a bioenergetics model: Examples for Age‐0 striped bass. Transactions of the American Fisheries Society, 122(5), 912–926. 10.1577/1548-8659(1993)122<0912:ssobia>2.3.co;2

[ece310974-bib-0035] Hasnain, S. S. , Minns, C. K. , & Shuter, B. J. (2010). Key ecological temperature metrics for Canadian freshwater fishes (Climate Change Research Report; CCRR‐17). Ministry of Natural Resources.

[ece310974-bib-0036] Hewett, S. W. , & Johnson, B. L. (1987). *A generalized bioenergetics model of fish growth for microcomputers*. University of Wisconsin, Sea Grant Institute, Technical Report WIS‐SG‐87‐245, Madison.

[ece310974-bib-0037] Hinke, J. T. , Watters, G. M. , Boehlert, G. W. , & Zedonis, P. (2005). Ocean habitat use in autumn by Chinook salmon in coastal waters of Oregon and California. Marine Ecology Progress Series, 285, 181–192. 10.3354/meps285181

[ece310974-bib-0038] Holden, J. P. , Connerton, M. J. , & Weidel, B. C. (2018). Hydroacoustic assessment of pelagic planktivores, 2017. NYSDEC Lake Ontario annual report 2017. New York State Department of Environmental Conservation.

[ece310974-bib-0039] Huey, R. B. , & Kingsolver, J. G. (2019). Climate warming, resource availability, and the metabolic meltdown of ectotherms. The American Naturalist, 194(6), E140–E150. 10.1086/705679 31738103

[ece310974-bib-0040] Huntsman, B. M. , Lynch, A. J. , & Caldwell, C. A. (2021). Interacting effects of density‐dependent and density‐independent factors on growth rates in southwestern cutthroat trout populations. Transactions of the American Fisheries Society, 150(5), 651–664. 10.1002/tafs.10319

[ece310974-bib-0041] Hussey, N. E. , Kessel, S. T. , Aarestrup, K. , Cooke, S. J. , Cowley, P. D. , Fisk, A. T. , Harcourt, R. G. , Holland, K. N. , Iverson, S. J. , Kocik, J. F. , Mills Flemming, J. E. , & Whoriskey, F. G. (2015). Aquatic animal telemetry: A panoramic window into the underwater world. Science (New York, N.Y.), 348(6240), 1255642. 10.1126/science.1255642 26068859

[ece310974-bib-0042] Hutchings, J. A. , & Myers, R. A. (1994). What can be learned from the collapse of a renewable resource? Atlantic cod, *Gadus Morhua*, of newfoundland and labrador. Canadian Journal of Fisheries and Aquatic Sciences, 51(9), 2126–2146. 10.1139/f94-214

[ece310974-bib-0043] Ivanova, S. V. , Johnson, T. B. , & Fisk, A. T. (2021). Movement ecology of a potamodromous top predator in a large lake: Synchrony and coexistence of distinct migratory patterns. Transactions of the American Fisheries Society, 1–13, 748–760. 10.1002/tafs.10325

[ece310974-bib-0044] Ivanova, S. V. , Johnson, T. B. , Metcalfe, B. , & Fisk, A. T. (2021). Spatial distribution of lake trout (*Salvelinus namaycush*) across seasonal thermal cycles in a large lake. Freshwater Biology, 66(4), 615–627. 10.1111/fwb.13665

[ece310974-bib-0045] Ivanova, S. V. , Larocque, S. M. , Fisk, A. T. , & Johnson, T. B. (2021). Spatiotemporal interactions of native and introduced salmonid top predators in a large lake: Implications for species restoration. Canadian Journal of Fisheries and Aquatic Sciences, 78(8), 1158–1167. 10.1139/cjfas-2020-0447

[ece310974-bib-0046] Ivanova, S. V. , Raby, G. , Johnson, T. B. , Larocque, S. M. , & Fisk, A. T. (2022). Effects of life stage on the spatial ecology of Chinook salmon (*Oncorhynchus tshawytscha*) during pelagic freshwater foraging. Fisheries Research, 254, 106395. 10.1016/j.fishres.2022.106395

[ece310974-bib-0047] Jacobs, G. R. , Madenjian, C. P. , Bunnell, D. B. , Warner, D. M. , & Claramunt, R. M. (2013). Chinook salmon foraging patterns in a changing Lake Michigan. Transactions of the American Fisheries Society, 142(2), 362–372. 10.1080/00028487.2012.739981

[ece310974-bib-0048] Jepsen, J. U. , Eide, N. E. , Prestrud, P. , & Jacobsen, L. B. (2002). The importance of prey distribution in habitat use by arctic foxes (*Alopex lagopus*). Canadian Journal of Zoology, 80(3), 418–429. 10.1139/z02-023

[ece310974-bib-0049] Johnson, R. C. , Beauchamp, D. A. , & Olden, J. D. (2023). Bioenergetics model for the nonnative redside shiner. Transactions of the American Fisheries Society, 152(1), 94–113. 10.1002/tafs.10392

[ece310974-bib-0050] Jordan, F. , Bartolini, M. , Nelson, C. , Patterson, P. E. , & Soulen, H. L. (1997). Risk of predation affects habitat selection by the pinfish *Lagodon rhomboides* (Linnaeus). Journal of Experimental Marine Biology and Ecology, 208(1–2), 45–56. 10.1016/S0022-0981(96)02656-1

[ece310974-bib-0051] Killen, S. S. (2014). Growth trajectory influences temperature preference in fish through an effect on metabolic rate. Journal of Animal Ecology, 83(6), 1513–1522. 10.1111/1365-2656.12244 24806155 PMC4277333

[ece310974-bib-0052] Kitchell, J. F. , Stewart, D. J. , & Weininger, D. (1977). Applications of a bioenergetics model to yellow perch (*Perca flavescens*) and walleye (*Stizostedion vitreum* vitreum). Journal of the Fisheries Research Board of Canada, 34(10), 1922–1935. 10.1139/f77-258

[ece310974-bib-0053] Kooijman, S. A. L. M. (2000). Dynamic energy and mass budgets in biological systems. Cambridge University Press. 10.1017/CBO9780511565403

[ece310974-bib-0054] Kraus, R. T. , Knight, C. T. , Farmer, T. M. , Gorman, A. M. , Collingsworth, P. D. , Warren, G. J. , Kocovsky, P. M. , & Conroy, J. D. (2015). Dynamic hypoxic zones in Lake Erie compress fish habitat, altering vulnerability to fishing gears. Canadian Journal of Fisheries and Aquatic Sciences, 72(6), 797–806. 10.1139/cjfas-2014-0517

[ece310974-bib-0055] Krueger, C. C. , Holbrook, C. M. , Binder, T. R. , Vandergoot, C. S. , Hayden, T. A. , Hondorp, D. W. , Nate, N. , Paige, K. , Riley, S. C. , Fisk, A. T. , & Cooke, S. J. (2018). Acoustic telemetry observation systems: Challenges encountered and overcome in the Laurentian great lakes. Canadian Journal of Fisheries and Aquatic Sciences, 75(10), 1755–1763. 10.1139/cjfas-2017-0406

[ece310974-bib-0056] Lynch, A. J. , Myers, B. J. E. , Chu, C. , Eby, L. A. , Falke, J. A. , Kovach, R. P. , Krabbenhoft, T. J. , Kwak, T. J. , Lyons, J. , Paukert, C. P. , & Whitney, J. E. (2016). Climate change effects on north American inland fish populations and assemblages. Fisheries, 41(7), 346–361. 10.1080/03632415.2016.1186016

[ece310974-bib-0057] Madenjian, C. P. , O'Connor, D. V. , & Nortrup, D. A. (2000). A new approach toward evaluation of fish bioenergetics models. Canadian Journal of Fisheries and Aquatic Sciences, 57(5), 1025–1032. 10.1139/f99-280

[ece310974-bib-0058] Matley, J. K. , Klinard, N. V. , Barbosa, A. P. , Martins, K. A. , Aspillaga, E. , Cooke, S. J. , Cowley, P. D. , Heupel, M. R. , Lowe, C. G. , Lowerre‐Barbieri, S. K. , Mitamura, H. , Moore, J.‐S. , Simpfendorfer, C. A. , Stokesbury, M. J. W. , Taylor, M. D. , Thorstad, E. B. , Vandergoot, C. S. , & Fisk, A. T. (2021). Global trends in aquatic animal tracking with acoustic telemetry. Trends in Ecology & Evolution, 37, 79–94. 10.1016/j.tree.2021.09.001 34563403

[ece310974-bib-0059] Mills, E. L. , Casselman, J. M. , Dermott, R. , Fitzsimons, J. D. , Gal, G. , Holeck, K. T. , Hoyle, J. A. , Johannsson, O. E. , Lantry, B. F. , Makarewicz, J. C. , Millard, E. S. , Munawar, I. F. , Munawar, M. , O'Gorman, R. , Owens, R. W. , Rudstam, L. G. , Schaner, T. , & Stewart, T. J. (2003). Lake Ontario: Food web dynamics in a changing ecosystem (1970–2000). Canadian Journal of Fisheries and Aquatic Sciences, 60, 471–490. 10.1139/F03-033

[ece310974-bib-0060] Morbey, Y. E. , Addison, P. , Shuter, B. J. , & Vascotto, K. (2006). Within‐population heterogeneity of habitat use by lake trout *Salvelinus namaycush* . Journal of Fish Biology, 69(6), 1675–1696. 10.1111/j.1095-8649.2006.01236.x

[ece310974-bib-0061] Mumby, J. A. , Larocque, S. M. , Johnson, T. B. , Stewart, T. J. , Fitzsimons, J. D. , Weidel, B. C. , Walsh, M. G. , Lantry, J. R. , Yuille, M. J. , & Fisk, A. T. (2018). Diet and trophic niche space and overlap of Lake Ontario salmonid species using stable isotopes and stomach contents. Journal of Great Lakes Research, 44(6), 1383–1392. 10.1016/j.jglr.2018.08.009

[ece310974-bib-0062] Nelson, J. S. (2006). Fishes of the world (4th ed.). John Wiley & Sons Inc.

[ece310974-bib-0063] Ney, J. J. (1990). Trophic economics in fisheries: Assessment of demand‐supply relationships between predators and prey. Reviews in Aquatic Sciences, 2, 55–81.

[ece310974-bib-0064] Ney, J. J. (1993). Bioenergetics modeling today: Growing pains on the cutting edge. Transactions of the American Fisheries Society, 122(5), 736–748. 10.1577/1548-8659(1993)122<0736:BMTGPO>2.3.CO;2

[ece310974-bib-0065] Olson, R. A. , Winter, J. D. , Nettles, D. C. , & Haynes, J. M. (1988). Resource partitioning in summer by salmonids in south‐Central Lake Ontario. Transactions of the American Fisheries Society, 117(6), 552–559. 10.1577/1548-8659(1988)

[ece310974-bib-0066] Plumb, J. M. , Blanchfield, P. J. , & Abrahams, M. V. (2014). A dynamic‐bioenergetics model to assess depth selection and reproductive growth by lake trout (*Salvelinus namaycush*). Oecologia, 175(2), 549–563. 10.1007/s00442-014-2934-6 24682254

[ece310974-bib-0067] Pyke, G. H. (1984). Optimal foraging theory: A critical review. Annual Review of Ecology and Systematics, 15(1), 523–575. 10.1146/annurev.es.15.110184.002515

[ece310974-bib-0068] Quinn, T. P. (2005). The behavior and ecology of Pacific Salmon and Trout. American Fisheries Society in Association with the University of British Columbia Press.

[ece310974-bib-0069] Raby, G. D. , Johnson, T. B. , Kessel, S. T. , Stewart, T. J. , & Fisk, A. T. (2017). A field test of the use of pop‐off data storage tags in freshwater fishes. Journal of Fish Biology, 91(6), 1623–1641. 10.1111/jfb.13476 29023720

[ece310974-bib-0070] Railsback, S. F. (2022). What we don't know about the effects of temperature on Salmonid growth. Transactions of the American Fisheries Society, 151(1), 3–12. 10.1002/tafs.10338

[ece310974-bib-0071] Railsback, S. F. , & Rose, K. A. (1999). Bioenergetics modeling of stream trout growth: Temperature and food consumption effects. Transactions of the American Fisheries Society, 128(2), 241–256. 10.1577/1548-8659(1999)128<0241:BMOSTG>2.0.CO;2

[ece310974-bib-0072] Reynolds, W. W. , & Casterlin, M. E. (1979). Behavioral thermoregulation and the ‘Final Preferendum’ paradigm. Integrative and Comparative Biology, 19(1), 211–224. 10.1093/icb/19.1.211

[ece310974-bib-0073] Ross, A. H. , & Nisbet, R. M. (1990). Dynamic models of growth and reproduction of the mussel *Mytilus edulis* L. Functional Ecology, 4(6), 777. 10.2307/2389444

[ece310974-bib-0074] Sonderegger, D. L. , Wang, H. , Clements, W. H. , & Noon, B. R. (2009). Using SiZer to detect thresholds in ecological data. Frontiers in Ecology and the Environment, 7, 190–195.

[ece310974-bib-0075] Spigarelli, S. A. , Thommes, M. M. , & Prepejchal, W. (1982). Feeding, growth, and fat deposition by Brown trout in constant and fluctuating temperatures. Transactions of the American Fisheries Society, 111, 199–209.

[ece310974-bib-0076] Stewart, D. J. , & Ibarra, M. (1991). Predation and production by Salmonine fishes in lake Michigan 1978–88. Canadian Journal of Fisheries and Aquatic Sciences, 48(5), 909–922. 10.1139/f91-107

[ece310974-bib-0077] Stewart, D. J. , Kitchell, J. F. , & Crowder, L. B. (1983). Forage fishes and their salmonid predators in lake Michigan. Transactions of the American Fisheries Society, 110, 751–763. 10.1577/1548-8659(1981)110<751

[ece310974-bib-0078] Stewart, D. J. , Weininger, D. , Rottiers, D. V. , & Edsall, T. A. (1983). An energetics model for lake trout, *Salvelinus namaycush*: Application to the lake Michigan population. Canadian Journal of Fisheries and Aquatic Sciences, 40(6), 681–698. 10.1139/f83-091

[ece310974-bib-0079] Tilman, D. (1980). Resources: A graphical‐mechanistic approach to competition and predation. The American Naturalist, 116(3), 362–393. 10.1086/283633

[ece310974-bib-0080] Trudel, M. , Tucker, S. , Morris, J. F. T. , Higgs, D. A. , & Welch, D. W. (2005). Indicators of energetic status in juvenile coho Salmon and Chinook Salmon. North American Journal of Fisheries Management, 25(1), 374–390. 10.1577/m04-018.1

[ece310974-bib-0084] Vander Zanden, V. , Jake, M. , Casselman, J. M. , & Rasmussen, J. B. (1999). Stable isotope evidence for the food web consequences of species invasions in lakes. Nature, 401(6752), 464–467. 10.1038/46762

[ece310974-bib-0081] Weidel, B. C. , Connerton, M. J. , & Holden, J. P. (2018). Bottom trawl assessment of Lake Ontario prey fishes. NYSDEC Lake Ontario annual report 2017. New York State Department of Environmental Conservation.

[ece310974-bib-0082] Wildhaber, M. L. , & Crowder, L. B. (1990). Testing a bioenergetics‐based habitat choice model: Bluegill (*Lepomis macrochirus*) responses to food availability and temperature. Canadian Journal of Fisheries and Aquatic Sciences, 47(9), 1664–1671. 10.1139/f90-190

[ece310974-bib-0083] Woolway, R. I. , Kraemer, B. M. , Lenters, J. D. , Merchant, C. J. , O'Reilly, C. M. , & Sharma, S. (2020). Global lake responses to climate change. Nature Reviews Earth and Environment, 1(8), 388–403. 10.1038/s43017-020-0067-5

