# Supplementary material for: Realized thermal niche approach eliminates temperature bias in bioenergetic model estimates
Source: Ecol Evol. 2024 Feb 14;14(2):e10974. doi: 10.1002/ece3.10974 (PMC10867506; doi:10.1002/ece3.10974)
Supplement: Supplementary file 1 — Appendix S1‐S2 [file ECE3-14-e10974-s001.pdf]

Appendix S1

Literature search was performed on the Web of Science with keywords search “bioenergetic” OR “bioenergetics”. The results (including article title, keywords and abstract) were further filtered for the keywords “fish” and “growth”, articles included for the years 2014 to 2023, and by relevant research areas, namely: Marine Freshwater Biology, Fisheries, Environmental Sciences Ecology, Physiology and Zoology; which yielded 383 papers. We performed a sub-search within the 383 results for the most common terms of “archival” and “telemetry” to quantify the number of studies that include either of these in the title, keywords or abstract. This produced 11 articles (i.e., ~3% that potentially used field data), all of which are shown below stating the type of temperature used in the models – of these 3% only 5 studies used observed temperature from the field.

For the remaining 372 articles, a random review of 30 articles revealed that approximately 60% were studies on wild fish that used assumed temperatures and the rest were lab-based or in aquaculture/hatchery setting.

**Study**	**Temperature (Observed Vs. Assumed/Inferred)/Relevance**
Brodie et al. 2016	Inferred/Yes
Dibble et al. 2023	NA/Not relevant: Expert panel results and only a mention
Eckmann et al. 2018	Observed/Yes
Cruz-Font et al. 2019	Inferred /Not relevant: theoretical study
Hahlbeck et al. 2022	Observed/Yes
Kraus et al. 2015	Inferred/Yes
Madenjian et al 2018	Observed/Yes
Peat et al. 2015	NA/Not relevant: Study only mentions bioenergetics model
Strom et al. 2023	Observed/Yes
Strople et al. 2018	Inferred/Yes
Westhoff et al. 2016	Observed/Yes

Appendix S2

Table S1 Number of observations per size class for lake trout and Chinook salmon used for calculating the energy density and equations.

**Size class**	**Lake trout**	**Chinook salmon**
**Juveniles**	1372	458
**Adults**	4394	932
**TOTAL**	**5766**	**1390**

Table S2 Prey energy density used for modelling in Fish Bioenergetics 4.

Abbreviations: NDMNRF – Ontario Ministry of Northern Development, Mines, Natural Resources, and Forestry; ED – energy density.

	**Alewife**	**Slimy sculpin**	**Deep-water sculpin**	**Rainbow smelt**	**Round goby**	**Bloater**
**Energy density (in J/g; source)**	4800 (NDMNRF)	5004 (Hondorp, Pothoven, and Brandt 2005)	3695 (Pothoven, Hondorp, and Nalepa 2011)	4500 (NDMNRF)	5800 (NDMNRF)	6,427 (Pothoven et al. 2012; Hondorp, Pothoven, and Brandt 2005)

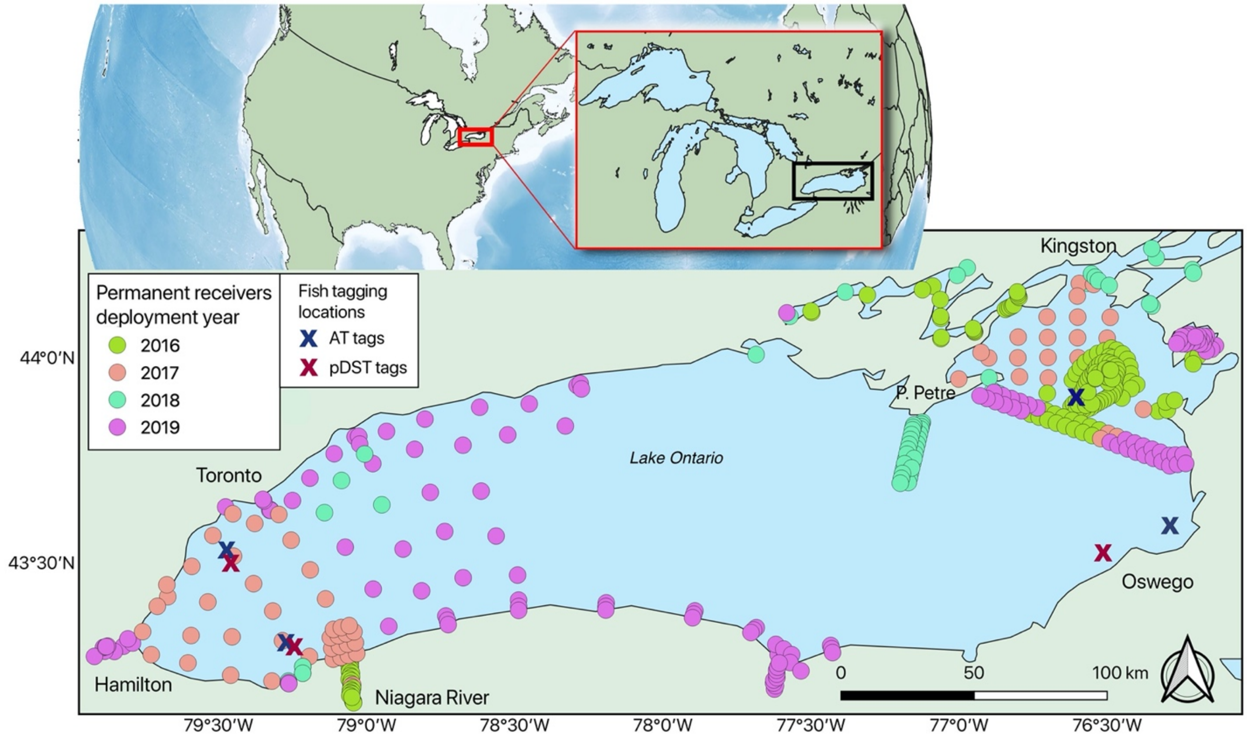

Fig. S1 Map of permanent receivers for the period 2016-2020 in Lake Ontario based on first year of deployment. Also shown are the tagging locations for Chinook salmon and lake trout with acoustic telemetry (AT) and pressure data storage tags (pDST). Base map sources: Esri, DeLorme, GEBCO, NOAA NGDC, National Geographic, HERE, Geonames.org and the GIS User Community (ESRI 2012), created using QGIS software.

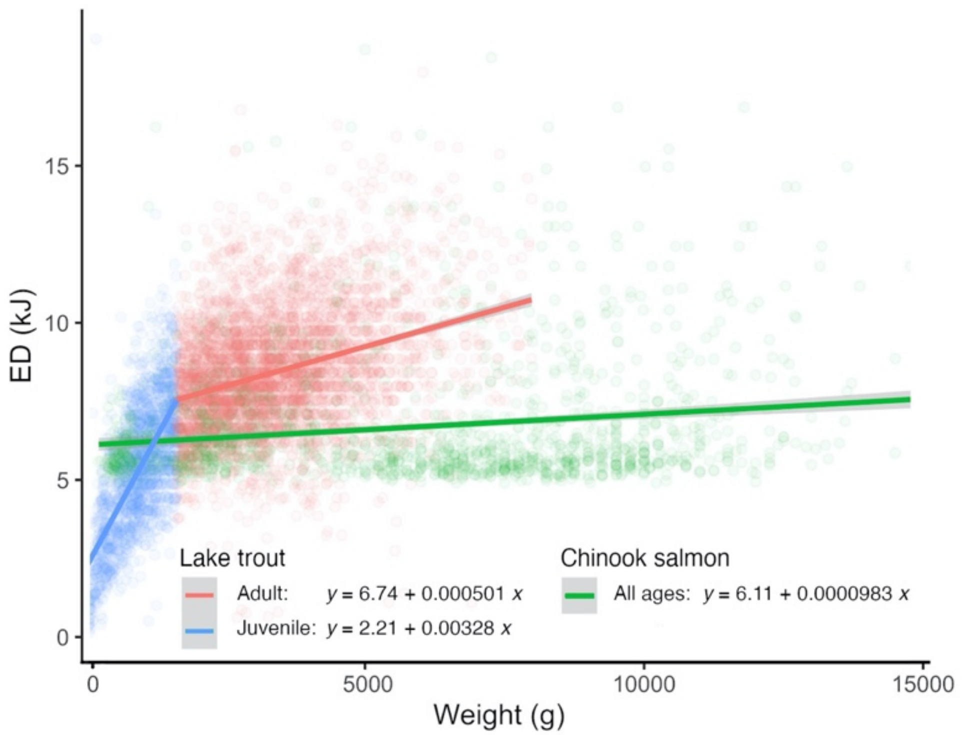

Fig. S2 Energy density and body weight relationships for Lake Ontario lake trout and Chinook salmon. Calculations were based on 5,766 lake trout and 1,390 Chinook salmon.

References:

ESRI. 2012. ‘Topographic [Basemap]. Scale Not Given’. World Topographic Map. 2012. http://www.arcgis.com/home/item.html?id=30e5fe3149c34df1ba922e6f5bbf808f.

Hondorp, Darryl W., Steven A. Pothoven, and Stephen B. Brandt. 2005. ‘ Influence of Diporeia Density on Diet Composition, Relative Abundance, and Energy Density of Planktivorous Fishes in Southeast Lake Michigan ’. *Transactions of the American Fisheries Society* 134 (3): 588–601. https://doi.org/10.1577/t04-107.1.

Pothoven, S. A., D. W. Hondorp, and T. F. Nalepa. 2011. ‘Declines in Deepwater Sculpin Myoxocephalus Thompsonii Energy Density Associated with the Disappearance of Diporeia Spp. in Lakes Huron and Michigan’. *Ecology of Freshwater Fish* 20 (1): 14–22. https://doi.org/10.1111/j.1600-0633.2010.00447.x.

Pothoven, Steven A., David B. Bunnell, Charles P. Madenjian, Owen T. Gorman, and Edward F. Roseman. 2012. ‘Energy Density of Bloaters in the Upper Great Lakes’. *Transactions of the American Fisheries Society* 141 (3): 772–80. https://doi.org/10.1080/00028487.2012.675911.
